# Random Convolutional Kernel Transform with Empirical Mode Decomposition for Classification of Insulators from Power Grid

**DOI:** 10.3390/s24041113

**Published:** 2024-02-08

**Authors:** Anne Carolina Rodrigues Klaar, Laio Oriel Seman, Viviana Cocco Mariani, Leandro dos Santos Coelho

**Affiliations:** 1 Graduate Program in Education, University of Planalto Catarinense, Lages 88509-900, Brazil; 2 Department of Automation and Systems Engineering, Federal University of Santa Catarina, Florianópolis 88040-535, Brazil; laio@ieee.org; 3 Mechanical Engineering Graduate Program, Pontifical Catholic University of Parana, Curitiba 80215-901, Brazil; viviana.mariani@pucpr.br; 4 Department of Electrical Engineering, Federal University of Parana, Curitiba 81530-000, Brazil; leandro.coelho@pucpr.br; 5 Industrial and Systems Engineering Graduate Program, Pontifical Catholic University of Parana, Curitiba 80215-901, Brazil

**Keywords:** electric power system, empirical mode decomposition, rocket algorithm, time series classification

## Abstract

The electrical energy supply relies on the satisfactory operation of insulators. The ultrasound recorded from insulators in different conditions has a time series output, which can be used to classify faulty insulators. The random convolutional kernel transform (Rocket) algorithms use convolutional filters to extract various features from the time series data. This paper proposes a combination of Rocket algorithms, machine learning classifiers, and empirical mode decomposition (EMD) methods, such as complete ensemble empirical mode decomposition with adaptive noise (CEEMDAN), empirical wavelet transform (EWT), and variational mode decomposition (VMD). The results show that the EMD methods, combined with MiniRocket, significantly improve the accuracy of logistic regression in insulator fault diagnosis. The proposed strategy achieves an accuracy of 0.992 using CEEMDAN, 0.995 with EWT, and 0.980 with VMD. These results highlight the potential of incorporating EMD methods in insulator failure detection models to enhance the safety and dependability of power systems.

## 1. Introduction

Electrical power grids form the backbone of modern society [1], and their components’ effective management and maintenance are of paramount importance [2]. Insulators play a critical role in ensuring the stability and reliability of these grids, as they serve as both mechanical supports for the wires and electrical potential insulation [3]. A degradation in an insulator’s characteristics can have severe consequences, leading to disruptive discharges, system failures, and compromised network dependability [4]. Therefore, it is crucial to develop robust and accurate methods for monitoring and assessing the performance of insulators [5].

Considering that insulators are responsible for keeping the power grid working by supporting and insulating the cables, the early identification of faulty insulators can assist the electric power utility in identifying where there are insulators that must be replaced [6]. The ultrasound signal can be recorded during power grid inspections, providing additional information for the inspection team. Therefore, the combination of an ultrasound signal and the proposed approach is an advanced way of identifying and mitigating faults in electrical power systems [7].

Inspections of the electrical system using radio-frequency-based techniques are increasingly being applied by power utilities since, before a fault occurs, partial discharges can emit light or noise that humans have difficulty identifying [8]. For this purpose, specific equipment, such as ultraviolet cameras, infrared cameras, and ultrasound detectors, are used [9]. Therefore, when there is a higher probability of failure, the maintenance team can take action in advance, improving the reliability of the electrical network [10].

Based on the ultrasound, analyzing time series data captured from the insulators during the inspections is a promising avenue for fault detection [11]. Ultrasound-based techniques have proven effective in identifying various insulator faults, providing valuable insights into their condition, and allowing for timely maintenance and replacement [12]. However, the accurate classification of these time series data remains a challenge. The major advantage of using ultrasound compared to leakage current detection [13], for example, is that the ultrasound does not need to be in direct contact with the network; thus, inspections can be performed with greater speed and less risk for the technical team [14].

Time series classification is a task that involves predicting a categorical label for a given time series dataset. This data are a sequence of observations collected over time. Time series classification aims to learn a model that can classify new time series based on past observations. Recent advances in time series classification methods have opened new possibilities for addressing this issue [15]. The accuracy and effectiveness of time series classification methods have recently undergone several types of substantial developments based on statistical models [16], machine learning [17], and deep learning [18] approaches. The random convolutional kernel transform (Rocket) algorithms [19], including MiniRocket [20] and MultiRocket [21], have attracted considerable attention from researchers due to their ability to efficiently and accurately process time series data.

Rocket is a kernel-based approach that uses random Fourier features to map data from time series into a feature space of high dimensionality. MiniRocket is a lightweight version of Rocket. MiniRocket is a faster and more memory-efficient method than Rocket, given that it only takes a small portion of the random Fourier features into account. Despite each time point being represented by many variables, MultiRocket is a variant of MiniRocket that is capable of handling multivariate time series classification challenges. Aiming to generate a shared feature representation for the multivariate time series, MultiRocket implements an innovative multivariate feature mapping technique that integrates the outputs from several univariate MiniRocket classifiers [22].

This paper proposes a novel approach, combining Rocket algorithms with machine learning classifiers to enhance insulator time series classification accuracy and efficacy based on ultrasound data. The contributions of this research are summarized below:

(i) An efficient classification framework that combines the advantages of Rocket approaches and machine learning models for the time series classification of medium voltage insulators is proposed, increasing classification accuracy and generalization capabilities.
(ii) The impact of integrating empirical mode decomposition methods with the proposed framework is shown, with significant improvements in classification accuracy.
(iii) Several classification algorithms are comprehensively compared to provide a benchmark for performance evaluation. This comparison will help engineers to select the most appropriate method for their specific insulator classification task, considering classification accuracy versus model complexity.

The proposed method was developed to identify the ultrasound signature of faulty insulators during inspections; based on this trained hybrid machine learning approach, the operator will obtain a classifier that has a higher chance of having an insulator that is not in good condition. Based on these measurements, predictive maintenance can be carried out to improve the power systems’ reliability.

The remainder of the work is presented as follows: The related works are briefly presented in Section 2. The description of the classification problem is detailed in Section 3. The fundamentals of the evaluated methods and the proposed approach are explained in Section 4. Section 5 discusses the classifier designs regarding performance evaluation and an analysis of the results, while Section 6 provides the main conclusions and suggestions for further research.

## 2. Related Works

The use of machine learning for fault estimation [23] is becoming popular for several tasks [24]. Several authors have explored the evaluation of time series for identifying and predicting insulator failures [25], as well as for other applications [26,27,28]. Klaar et al. [29] used the empirical wavelet transform for denoising in a hypertuned long short-term memory (LSTM) for fault prediction in insulators considering a sequence-to-sequence problem.

The prediction regarded leakage current, similar to the method presented by Sopelsa Neto et al. [30] and Medeiros et al. [31], where several models were explored for this task. As presented by Zhang et al. [32] the long-term monitoring of electrical systems is important to ensure their performance and reliability over time. As presented in [33] there is a trend of adopting energy harvesting techniques for fault diagnosis.

Insulator classification is a task that other researchers have covered, considering that these components need to be in good condition to keep the electrical power system running. Tao et al. [34], Tan [35], Mano, Tomohiro, and Ohtsuki [36], and She et al. [37] considered convolutional neural networks (CNNs) for image classification based on aerial images of insulators in different conditions. According to these works [34,35,36,37], the application of CNN is well known to be a promising alternative when images of inspections are analyzed.

This paper considers the CNNs to analyze the ultrasound signal, which is an innovative way of evaluating the time series of the ultrasound detected from insulators in different conditions. The use of CNNs for time series classification proposed here is an outstanding solution in this field since the majority of the research considers images from visual inspections.

### 2.1. Visual Inspections and Classification

To automate visual inspection tasks, Prates et al. [38] suggested applying CNNs to recognize flaws and different insulators in overhead power distribution lines (OPDLs). More than 2500 photos obtained from a studio and a realistic OPDL were used to train the model. Multi-task learning was also employed to enhance fault detection performance by predicting the insulator class. Also, based on images, in [39], a new hybrid method is proposed, which combines object detection to CNNs for classification.

The you only look once (YOLO) deep learning neural network model using the unmanned aerial vehicle has been presented in the work of Sadykova et al. [40] as an effective technique for detecting high-voltage insulators. The purpose is to provide a real-time classification of insulator conditions while avoiding expensive manual inspections that involve traveling across a wide area in adverse weather. The technique uses a training set size of 56,000 image samples and data augmentation to prevent overfitting. The experimental findings show how well the proposed method works for accurately determining insulators and assessing their surface conditions for the presence of ice, snow, and water through different classifiers. Also, in [41], a hybrid version of YOLO is proposed for inspections of the power grids.

Aiming to monitor the condition of equipment for high-voltage power stations, Mitiche et al. [42] addressed the use of bispectrum representations as complex input features in complex-valued deep CNNs. This approach achieved excellent classification accuracy for discharge signals. An automated inspection system that uses computer vision to gauge erosion in silicone rubber samples was presented by Ibrahim et al. [3]. Using the International Electrotechnical Commission (IEC-60587 [43]) standard to describe failure, the system was intended to classify samples into one of three groups based on the level of erosion. The suggested system compared the performance of ANNs, applying feature extraction methods and pre-processing approaches.

A novel model based on feature pyramid neural networks and an adaptive threshold algorithm with line detection, image rotation, and vertical projection data, applied to insulator fault detection in transmission lines, was proposed by Zhao et al. [44]. Singh et al. [45] presented an evaluation of infrared thermal images; their method computes several features from the segmented region of interest and utilizes a Gaussian kernel SVM to classify the insulator faults. A robust methodology based on deep learning and uncertainty detection for automatic insulator fault inspection, using aerial images, was approached by Dai [46]. The bounding box prediction was improved, and the detection robustness was enhanced using the predicted uncertainty scores.

### 2.2. Time Series and Machine Learning

The problem of interpreting leakage current measurements for overhead insulator condition monitoring due to the intermittent harmonic content of the supply voltage was presented by Ghosh et al. [47]. To monitor leakage current in the presence of voltage harmonics, the study suggested applying the instant value of the time integral of the leakage current as a low-sensitivity parameter. The study demonstrated that changes in system voltage’s harmonic content significantly impact the harmonic properties of the leakage current. The suggested measuring method was tested and validated using experimental data that were captured in the lab and integrated into an online measurement tool that was evaluated in the lab.

The problem of appropriately simulating the flashover phenomena in contaminated insulators was approached by Belhouchet et al. [48]. This issue is made more challenging by the complexity of determining the arc constants generated in dry bands when the electrical voltage goes beyond critical levels. Using data from artificially contaminated insulator experiments, the authors suggest a strategy for optimization based on genetic algorithms and artificial neural networks (ANN) to identify the arc constants and dielectric properties of the surface. The research, which used a generalized pollution flashover model, observed that the inverse connection of flashover voltage and leakage current was validated by the optimized mathematical model’s realistic simulation of the experimental data.

A method for predicting line trip defects in power systems that combine a support vector machine and LSTM networks was suggested by Zhang et al. [49]. The suggested approach addresses the shortcomings of existing approaches based on the activities that are carried out to preserve relays and electrical components. In order to acquire the final prediction results, the support vector machine is used for classification and the LSTM networks are employed to capture the temporal aspects of multi-sourced data. The LSTM is suggested for time series with high nonlinearities [50] and can be further improved by using the attention mechanism [51].

Polisetty et al. [52] concentrated on the significance of keeping a close watch on outside insulation systems to preserve the integrity of substations and overhead transmission and distribution lines. The study used an ANN and a commercial acoustic sensor to classify the electrical discharge patterns in external insulating systems. The ANN was then expanded to incorporate three different types of flaws on outdoor ceramic insulators and distinguish between five frequent discharges of electricity produced under controlled settings. The investigation successfully identified approximately 85% of the controlled samples.

A new method for insulator condition monitoring based on meteorological and environmental information was suggested by De Santos and Sanz-Bobi [53]. The method combined the random under-sampling technique to estimate important condition indicators with an adaptive boosting algorithm (RUSBoost). The proposed method was compared with other algorithms at France’s 245 kV test station. The findings indicate that RUSBoost outperformed the competitors’ algorithms, rating highly in the estimation of insulator conditions. Advanced hybrid methods were applied by several researchers [54,55,56], and the idea of combining techniques helps the model by using the advantages of more than one approach.

A knowledge-based optimization approach to deal with the challenge of determining the optimal process settings for manufacturing medium voltage insulators was proposed by Kong et al. [57]. Their method utilized historical approximations produced during the optimization process to enhance the accuracy of the gradient estimates and to adjust the size of the iteration step. Their approach reduced the cost and improved the quality control efficiency for insulators, which is crucial for their efficient production and confident operation. Models based on the ensemble approach are promising as they usually need less computational effort compared to deep learning [58].

The adoption of deep learning algorithms for the condition monitoring of high voltage equipment in electrical power systems was reviewed by Mantach et al. [59]. Contrasting conventional machine learning approaches, deep learning combines feature extraction with the learning stage and uses raw data as input. This paper included contemporary research on deep learning approaches for monitoring high-voltage equipment, including gas-insulated switchgear, transformers, cables, rotating machines, and outside insulators.

A novel approach to monitoring the pollutant insulator discharge mode in high-voltage lines by combining auditory emission signals with a one-dimensional CNN structure (1D-CNN) was presented by Hao et al. [60]. The procedure includes data collection in a lab, accompanied by the use of 1D-CNN to reduce the dimensionality of the signal samples and extract features. With a recognition rate of over 99.84%, the model successfully replaces the need for human data preparation in conventional monitoring approaches and may be used to carry out monitoring tasks for the pollution insulator discharge mode.

A CNN bidirectional LSTM, named CNN-Bi-LSTM neural network design with hyperparameter optimization, used to classify leakage current levels according to sequential weather factors and insulator data, was evaluated by Nguyen et al. [61]. The CNN-Bi-LSTM was employed in real-time monitoring services to improve the operations of the TaiPower electric utility in Taiwan. On the other hand, a CNN-LSTM neural network with hyperparameter tuning for categorizing the leakage/discharge current on a web-based service was evaluated by Tham and Cho [62]. Leakage current surge and weather data are used as input parameters in four different models to predict leakage current classification.

### 2.3. Ultrasound Detector

Using ultrasound to classify the condition of the insulators of medium voltage power grids, Stefenon et al. [63] proposed using the echo state network. They showed that identifying a specific condition, such as drilling, is easier than performing a multiclassification. They highlighted that the broken and drilling insulators have more partial discharges than contaminated or clean insulators, making it possible to obtain classification results with over 99% accuracy when these conditions are evaluated. In this evaluation, the echo state network was more promising than the support vector machine (SVM) or multilayer perception.

Ferreira et al. [64] proposed a method for calculating electrical insulator pollution using ultrasonic noise. The audio was reduced using the spectral subband centroid energy vectors’ algorithm before being input into an artificial neural network that can distinguish between different degrees of pollution. Their method was applied to process ultrasonic sounds from different types of electrical equipment given to multiple forms of pollution. In [65], the contamination of insulators was evaluated using deep learning.

Concerning an evaluation of the time series to predict the increase in faults in the power supply system, Branco et al. [66] presented a study of the number of faults that occurred over the year. The failures could be related to climatic variations. Depending on the season, more failures can occur, especially in this study, where there is a rainy season, increasing the probability of failure [67]. A highlight that was presented in this research was the use of wavelet transform to mitigate the impact of unrepresentative variations. This technique can be used in chaotic time series, such as ultrasound, which are studied to detect failures in power utilities [68].

The study of ultrasound has been explored by several authors [69,70,71,72], and can be applied to classification, as presented in this paper. Considering an experiment under medium voltage, the ultrasound equipment is employed to define insulator patterns under different conditions, as will be explained in detail in the next section.

## 3. Insulators Ultrasound Measurement

**Figure 1:**
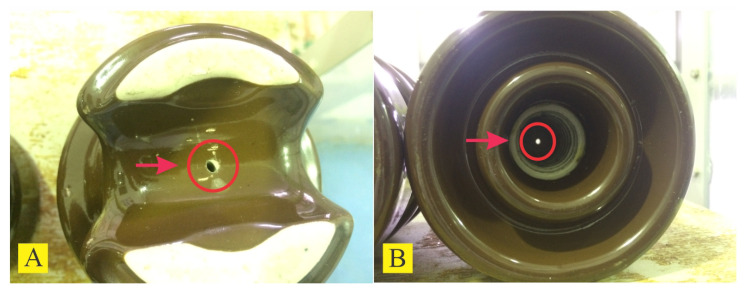
Insulator drilled with a bench drill to simulate a perforation caused by an electric discharge: (**A**) top view; (**B**) bottom view.

This section provides a detailed account of the classification problem and explains the experiment performed in the high-voltage laboratory presented in Figure 1. The experiment involves applying a voltage of 7.95 kV phase-to-ground to the insulators, equivalent to 13.8 kV phase-to-phase in the power system, the electrical potential used in the considered distribution branch located in southern Brazil.

This paper considered three conditions: an insulator in good condition, an artificially contaminated insulator, and a drilled insulator. The insulators are pin-type profiles, class 15 kV, from the Germer manufacturer. These insulator profiles are commonly installed in conventional distribution power grids in rural southern Brazil, which are exposed to organic contamination from unpaved roads and saline contamination when close to the coast [73].

To simulate the drilling caused by lightning, a perforation was performed on the top of insulators using a bench drill. Figure 1A presents the top view of the drilling, and Figure 1B shows the bottom view, where the fixation pin is attached. This problem is less present in the distribution grid because the perforations can occur underneath the mooring. Perforation is more common in polymeric insulators, where the temperature required for carbonization is lower than that in glass insulators [74].

The contamination over the insulator surface is an issue that increases the conductivity of the surface, leading to a higher leakage current and possible flashovers [75]. The flashovers mainly occur in bad weather conditions, making it challenging to identify the exact location of the fault during inspections. When lightning strikes the electrical power grid, perforations or boundary discharges might occur, resulting in a higher risk of irreversible failures, for which corrective maintenance is required [76].

To simulate the contamination on the insulators, the solid layer procedure presented by NBR 10621/2017 [77] (Brazilian Standard: high-voltage insulators to be used in alternating current systems—artificial pollution tests) was followed, based on IEC-507 [78] (artificial pollution tests on high-voltage insulators to be used in alternating current systems). The contaminants that were considered were kaolin and sodium chloride. The NBR 10621/2017 [77] standard determines the tolerable performance of porcelain or glass insulators for outdoor applications [79].

**Figure 2:**
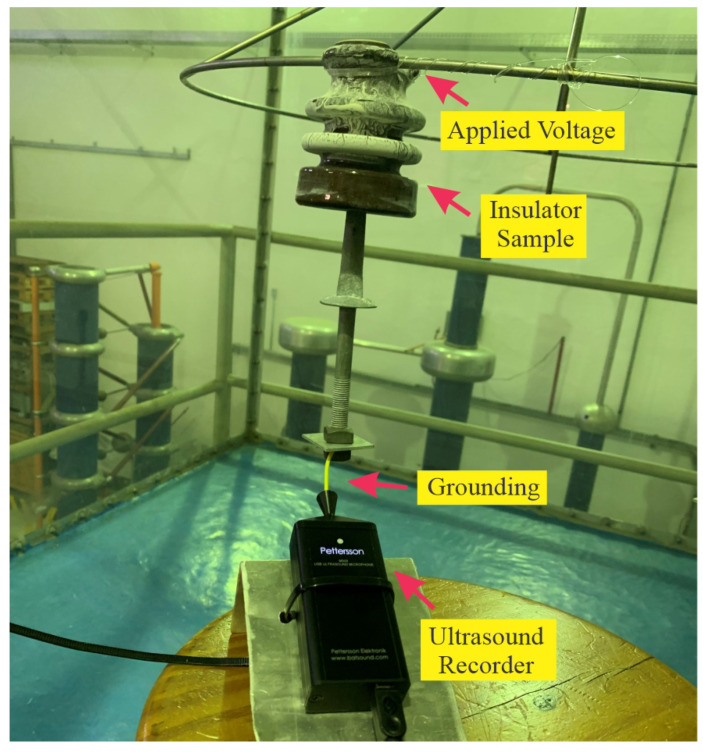
High-voltage applied experiment under controlled conditions.

The experiment was conducted inside an acrylic chamber since the ultrasound detector is sensitive to noise from external sources. The ultrasound detector was set at a distance of 0.4 m from the insulators (see Figure 2), and recorded noise signals with a maximum frequency of 500 kHz; this distance was fixed for comparative purposes. In the distribution grids, the operator may face scenarios with varying relief, which may result in a greater need for measurement. The difficulty in reaching the grid is one of the significant challenges in inspections of power distribution networks carried out by the electric utility company [80].

To mitigate the interference from partial discharges resulting from the mooring of the insulator, the fixing was carried out with non-conductive materials [81]. The chamber held two affixed insulators, and voltage was applied to these insulators while the ground contained an equal reference. The ground was attached only to the insulator under evaluation to prevent one insulator from affecting the other. An M500 model from Petterson recorded the ultrasonic signal. The conductivity of the water used to spray the samples during the experiment was 56 kg/m^3^, which corresponds to a medium–high contamination level according to IEC-507.

**Figure 3:**
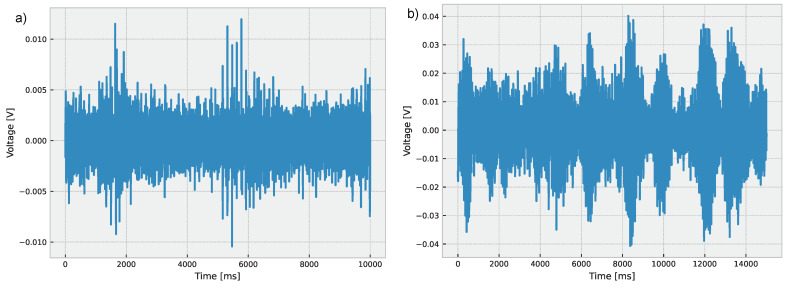
Ultrasound recorded signal: (**a**) normal; (**b**) fault.

As partial discharge typically occurs over the frequency range from 10 kHz to 210 kHz, a comprehensive assessment of more than 50 times the base frequency ensures that all frequencies beyond 10 kHz are captured under a single wave cycle. In addition to the 500 kHz frequency rate, to ensure that the signal was properly recorded for a sufficient length of time, the data log was held for 50 s. After the signal was saved, a total of 1 $\times \;10^{5}$ records were considered for a comprehensive assessment. Figure 3 presents an example of the signal recorded by the ultrasound equipment.

During the experiments, the faulty insulators were subjected to a voltage equal to that of the electrical power network in which they are employed. This condition does not result in a flashover, since this fault usually occurs when, in addition to the contamination, there is high humudity. Considering that the focus of this paper is to identify faulty insulators before failure occurs under regular environmental conditions, there is no flashover and the ultrasound is not measured under this condition.

## 4. Methodology

**Figure 4:**
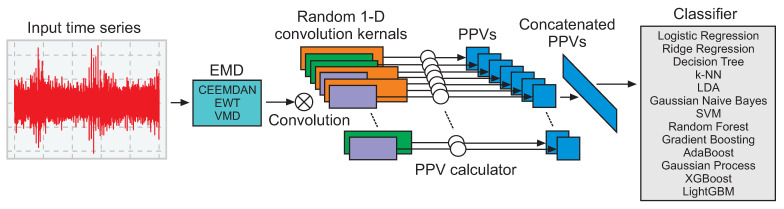
Architecture of the proposed model.

The model proposed in this paper combines empirical mode decomposition methods with random convolutional kernel transform models and state-of-the-art classifiers to obtain a hybrid architecture, as presented in Figure 4, which is explained in this section. The input signal is based on a time series measured by ultrasound during high-voltage experiments considering insulators under different conditions, as explained in the previous section.

A time series is a sequence of points of information collected over time, typically at fixed intervals. The classification of time series is related to the development of models to classify time series data into predetermined categories based on their patterns and characteristics over time. The Rocket [19], MiniRocket [20], and MultiRocket [21] algorithms have been widely evaluated for time series classification tasks.

The fundamental concept of Rocket methods is to obtain features from time series data and use these features to train a classifier. These models use convolutional kernels to transform the time series data into features, which are then used for classification [82]. Given a time series $x=x_{1},x_{2},\ldots ,x_{T}$, these algorithms compute features such as the maximum value (Max) and the proportion of positive values (PPV) for each of the *k* convolutional kernels. The convolutional operation for a kernel $\phi =\phi_{1},\phi_{2},\ldots ,\phi_{m}$ can be expressed as follows:

(1) $$z_{i}=\left(x*\phi \right)i=\sum {j=1}^{m}x_{i+j-1}\phi_{j},$$

where ∗ denotes the convolutional operation, $z_{i}$ is the output of the convolution, and *m* is the kernel length. The Max and PPV features are computed as follows:

(2) $$\mathrm{Max}=\max_{1\leq i\leq T-m+1}z_{i},$$

(3) $$\mathrm{PPV}=\frac{1}{T-m+1}\sum_{i=1}^{T-m+1}1\left(z_{i}>0\right),$$

where 1(·) is the indicator function. The extracted features are then used to train a linear classifier for time series classification.

MiniRocket distinguishes itself from Rocket by computing features using a fixed set of *k* convolutional kernels with a shorter kernel length, resulting in greater computational efficiency and refining the convolutional process by introducing alterations to the kernels [83]. The MiniRocket transform calculates the Max and PPV features for each *k* fixed convolutional kernel. By leveraging a fixed set of kernels with shorter kernel lengths, MiniRocket significantly diminishes the computational effort while retaining a competitive performance in time series classification tasks [84].

The MultiRocket algorithm extends the Rocket framework by incorporating multiple pooling operators and transformations to enhance the diversity of the generated features. MultiRocket employs first-order differences to transform the primary input series, thereby creating an alternative representation and processing the raw input series. Both representations undergo the application of convolutions, and the processing of the convolution outputs is executed using four distinct pooling operators. The integration of additional procedures expands the range of features and improves the algorithm’s performance [21].

### 4.1. Empirical Mode Decomposition

For time series decomposition, feature extraction, and noise reduction, the empirical mode decomposition (EMD) methods are applied [85]. The variations in the EMD include the complete ensemble empirical mode decomposition with adaptive noise (CEEMDAN) [86], empirical wavelet transform (EWT) [87], and variational mode decomposition (VMD) [88]. These methods are advanced signal processing techniques that aim to decompose a given time series into a finite set of components, with each representing an intrinsic mode function (IMF) [89] or oscillatory mode.

EMD is a data-driven method that decomposes non-linear time series in a set of IMFs [90]. The main idea behind EMD is the so-called sifting process, which iteratively extracts IMFs by identifying local extrema and fitting envelopes using cubic spline interpolation. Given a time series x(t), the sifting process begins with the identification of all the local maxima and minima. Next, the upper and lower envelopes are created by the interpolation of the local maxima and minima, employing cubic spline interpolation. The mean of the envelopes is then calculated as follows:

(4) $$m\left(t\right)=\frac{1}{2}\left(e_{\mathrm{upper}}\left(t\right)+e_{\mathrm{lower}}\left(t\right)\right),$$

where $e_{\mathrm{upper}}\left(t\right)$ is the upper envelope and the $e_{\mathrm{lower}}\left(t\right)$ is the lower envelope [91].

The difference between the original signal and the mean is considered a candidate IMF:

(5) c(t)=x(t)−m(t),

and this process is repeated on the IMF until it meets the predefined stopping criterion. Then, it is applied to the residual signal until all IMFs are extracted.

The EWT involves the decomposition of a given signal in oscillatory modes with varying scales and frequencies [92]. The EWT algorithm produces a collection of *n* non-linear functions, known as IMFs, from the signal x(t) and a wavelet mother function ψ(t). The process of generating these IMFs is outlined in Algorithm 1.
**Algorithm 1:** EWT
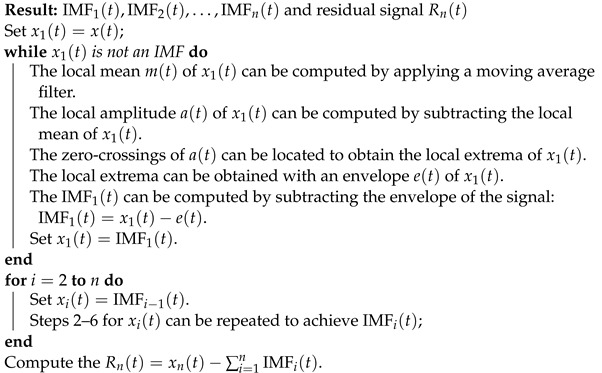


Once the set of IMFs is obtained, EWT employs a Fourier transform to each IMF to produce a set of *n* spectrograms, which are utilized to visualize the time-frequency information of the signal [93]. The EWT has the following expression:

$$\begin{array}{cc} x(t) & =\sum_{i=1}^{n}{\mathrm{IMF}}_{i}\left(t\right)+R_{n}\left(t\right)\\ {\mathrm{IMF}}_{i}\left(t\right) & =\int x\left(\tau \right)h_{i}\left(\tau -t\right)d\tau \end{array}$$

where $h_{i}\left(\tau \right)$ is the *i*th filter, set as the convolution of the scaling function φ(t), and the ψ(t) is scaled by a factor of $2^{i}$:

(6) $$\begin{array}{cc} \; & h_{i}\left(\tau \right)=2^{i}\varphi \left(2^{i}\tau \right)\psi \left(2^{i}\tau \right). \end{array}$$

The EWT combines the concepts of EMD and wavelet transform. The main idea of EWT is to decompose the signal in a set of oscillatory modes using an adaptive filter bank. The filter bank is designed based on the signal’s time-frequency content, estimated by the continuous wavelet transform [94]. The EWT decomposition is as follows:

(7) $$x\left(t\right)=\sum_{i=1}^{N}w_{i}\left(t\right)+r\left(t\right),$$

where $w_{i}\left(t\right)$ are the wavelet components, *N* is the number of modes, and r(t) is the residual.

VMD is another decomposition technique that formulates the extraction of IMFs as a constrained variational problem. VMD decomposes the time series in a set of band-limited IMFs by minimizing the cost function that balances the compactness of the frequency spectrum and the smoothness of the time-domain signal [95]. The VMD optimization problem can be written as follows:

(8) $$\begin{array}{cc} \; & \min_{u_{k}\left(t\right),\omega_{k}}\sum_{k=1}^{K}\int {\left|\partial_{t}\left(\frac{u_{k}\left(t\right)}{1+j\omega_{k}t}\right)\right|}^{2}dt \end{array}$$

(9) $$\begin{array}{cc} \; & s.t.\;\;x\left(t\right)=\sum_{k=1}^{K}u_{k}\left(t\right) \end{array}$$

where $u_{k}\left(t\right)$ are the mode functions, *K* is the number of modes, and $\omega_{k}$ are the center frequencies of the modes.

### 4.2. Classification Methods

To evaluate the effectiveness of Rocket methods, including MiniRocket and MultiRocket, in classifying faults in insulators, a comprehensive analysis is conducted by combining these algorithms with various classifiers mentioned above. This experimental design aims to determine the best-suited combination of Rocket techniques and classification methods, ultimately enhancing insulator fault detection accuracy.

**Logistic Regression**: Logistic regression, a prevalent linear technique employed for classification, utilizes a logistic function to model the probability of a specific class or event [96]. The following equation represents the logistic function:

(10) $$\begin{array}{c} P\left(y=1|x\right)=\frac{1}{1+e^{-(\beta_{0}+\beta_{1}x)}} \end{array}$$

**Ridge Regression**: Ridge regression, also known as Tikhonov regularization, is a linear regression technique incorporating an L2 regularization term to address the issue of multicollinearity and improve the generalization of the model [97]. This is particularly useful when there are highly correlated features. The objective function for ridge regression can be written as follows:

(11) $$\begin{array}{c} L\left(w,b\right)=\sum_{i=1}^{n}{\left(y_{i}-\left(w^{T}x_{i}+b\right)\right)}^{2}+\lambda {\left|w\right|}^{2} \end{array}$$

where w is the weight vector, *b* is the bias term, $y_{i}$ and $x_{i}$ are the true label and the feature vector for the *i*-th instance, respectively, and λ is the regularization parameter that controls the trade-off between model complexity and the goodness of fit. The regularization term, ${\lambda |w|}^{2}$, discourages the model from assigning large weights to the features, leading to a more stable and robust solution.

**Decision Tree**: The decision tree classifier, a non-parametric, hierarchical model, recursively partitions the input space into discrete regions according to feature values. The decision rules are derived by minimizing the impurity of the resultant nodes, which can be quantified utilizing metrics such as Gini impurity or entropy [98].

The architecture of the classifier is built in the form of a tree structure, where each internal node represents a feature or attribute, each branch represents a decision rule, and each leaf node represents a class label or a decision. According to Mishra et al. [99], the architecture can be further improved using clustering techniques.

**k-NN**: The k-nearest neighbors (k-NN) classifier, a non-parametric, instance-based learning algorithm, classifies novel instances based on the majority class of their *k* nearest neighbors. The distance metric and the value of *k* are crucial to the algorithm’s performance. Since it is a classification problem, employing an odd *k* is more advantageous, avoiding draws [100]. For this task, the weighted mode is denoted by the following:

(12) $$\begin{array}{c} \gamma_{t}=\arg\max_{c\in Y}\sum_{i=1}^{k}\omega_{i}I\left(c,\gamma_{i}\right) \end{array}$$

where,

(13) $$\omega_{i}=\frac{1}{d(x_{t},x_{i})},$$

I(a,b) returns 1 if a=b [101]. $\gamma_{i}$ is the class of the $x_{i}$ example associated with the $\omega_{i}$ weight, and *c* is the class with the best-weighted mode. To calculate the neighbors the Euclidean, cosine, correlation, chebychev, city block, spearman, standardized Euclidean, Minkowski, and Mahalanobis distances methods can be applied [102].

**LDA**: Linear discriminant analysis (LDA), a technique utilized for dimensionality reduction and classification, identifies the linear combination of features that optimally separates distinct classes by maximizing the dispersion between classes and minimizing the dispersion within a class [103]:

(14) $$\begin{array}{c} J\left(w\right)=\frac{w^{T}S_{B}w}{w^{T}S_{W}w} \end{array}$$

where $S_{B}$ and $S_{W}$ represent the between-class and within-class scatter matrix, respectively.

**Gaussian Naive Bayes**: Gaussian Naive Bayes is a classification algorithm that is based on Bayes’ theorem [104], assuming the features are conditionally independent and follow a Gaussian distribution:

(15) $$\begin{array}{c} P\left(A|B\right)=\frac{P(B|A)P(A)}{P(B)} \end{array}$$

where *A* and *B* are events or variables. The Gaussian Naive Bayes assumes that the features in the dataset are normally distributed and that they are independent of each other.

**SVM**: The support vector machine (SVM) classifier endeavors to identify the optimal separating hyperplane between classes [105]. Its performance is governed by the kernel function and regularization parameter *C*:

(16) $$\begin{array}{cc} \; & \min_{w,b,\xi }\frac{1}{2}w^{T}w+C\sum_{i=1}^{n}\xi_{i} \end{array}$$

(17) $$\begin{array}{cc} \; & s.t.\;\;y_{i}\left(w^{T}x_{i}+b\right)\geq 1-\xi_{i} \end{array}$$

(18) $$\begin{array}{cc} \; & \xi_{i}\geq 0. \end{array}$$

**Random Forest**: An ensemble learning methodology constructs multiple decision trees and amalgamates their outputs via majority voting [106]. The operator regulates the number of trees (*T*) and their maximum depth. Let X be the set of input features and Y be the set of output classes. The random forest classifier consists of *T* decision trees, ${h_{t}\left(x\right)}_{t=1}^{T}$, with each grown to a maximum depth. Each tree is created by a randomly sampled subset of the train data, typically using a replacement (i.e., bootstrapped samples), and a random subset of input features at each split. The random forest classifier is provided using the following definition:

(19) $$\begin{array}{c} H\left(x\right)=\arg\max_{c\in Y}\sum_{t=1}^{T}I\left(c,h_{t}\left(x\right)\right), \end{array}$$

where H(x) represents the final classification, again I(a,b) returns 1 if a=b, and 0 otherwise, and $h_{t}\left(x\right)$ is the output of the *t*-th decision tree for input *x*.

**Gradient Boosting**: The gradient boosting classifier, an ensemble learning technique, sequentially builds weak learners, with each learner rectifying the errors committed by the preceding one [107]:

(20) $$\begin{array}{c} F_{m}\left(x\right)=F_{m-1}\left(x\right)+\rho_{m}h_{m}\left(x\right) \end{array}$$

where $F_{m}\left(x\right)$ denotes the boosted model at step *m*, $h_{m}\left(x\right)$ signifies the weak learner, and $\rho_{m}$ represents the step size. The gradient boosting method has also been utilized for prediction by various authors [108,109,110].

**AdaBoost**: Adaptive boosting (AdaBoost) classifier, an adaptive boosting technique, combines weak learners to form a robust classifier, with each learner weighted based on its accuracy [111]. The algorithm updates the weights of the training instances at each iteration, assigning greater importance to misclassified instances:

(21) $$\begin{array}{c} D_{t+1}\left(i\right)=\frac{D_{t}\left(i\right)e^{-\alpha_{t}y_{i}h_{t}\left(x_{i}\right)}}{Z_{t}} \end{array}$$

where $D_{t}\left(i\right)$ is the weight of instance *i* at iteration *t*, $h_{t}\left(x_{i}\right)$ is the prediction, $y_{i}$ is the true label, $\alpha_{t}$ is the weight of the weak learner, and $Z_{t}$ is the normalization factor.

**Gaussian Process**: Gaussian process classifier, a Bayesian, non-parametric model, employs a Gaussian process prior over the function space and yields probabilistic classification results [112]. This is determined by a mean m(x) and a covariance function $k(x,x^{\prime })$:

(22) $$\begin{array}{c} f\left(x\right)∼\mathrm{GP}(m\left(x\right),k\left(x,x^{\prime }\right)) \end{array}$$

**XGBoost**: The extreme gradient boosting (XGBoost) algorithm is a highly efficient and scalable tree-based ensemble learning model, designed for both classification and forecasting problems [113]. It is an extension of the gradient boosting algorithm, employing advanced regularization techniques to improve the model’s generalization and control overfitting. XGBoost optimizes the following objective function:

(23) $$\begin{array}{c} L\left(\phi \right)=\sum_{i=1}^{n}l\left(y_{i},\hat{y}i\right)+\sum_{j=1}^{K}\Omega \left(f_{j}\right) \end{array}$$

where ϕ represents the model parameters, $l(y_{i},{\hat{y}}_{i})$ denotes the loss function comparing the true label $y_{i}$ and the forecasted label ${\hat{y}}_{i}$, and $\Omega (f_{j})$ is the regularization term for the *j*-th tree. The regularization term comprises the tree complexity, measured by the number of leaves *T*, and the L2-norm of the leaf scores *w*:

(24) $$\begin{array}{c} \Omega \left(f\right)=\gamma T+\frac{1}{2}\lambda {\left|w\right|}^{2} \end{array}$$

The algorithm employs second-order gradient information (Hessian) and the first-order gradient to update the model, making the learning process more accurate and faster. Furthermore, it utilizes column block and sparsity-aware techniques to efficiently handle sparse data and parallelize the tree construction process, enabling it to tackle large-scale datasets efficiently [114].

**LightGBM**: The light gradient boosting method (LightGBM), a boosting framework, leverages tree-based learning algorithms and is designed to be efficient and scalable for large datasets [115]. It adopts gradient-based one-sided sampling and exclusive feature bundling to expedite training and diminish memory usage.

The Rocket algorithm is applied considering Equations (1)–(3). For the application of this method, the time series is denoised using the EMD methods, which can be defined by Equations (4)–(9). Considering a denoised signal, the classifiers are evaluated; these classifiers are explained considering Equations (10)–(24).

A limitation of the application of the proposed method is that other signals measured during the inspection may result in interference at high frequencies; therefore, a specialist operator needs to perform the measurements to ensure that the signal is correctly recorded. This means that the gain of the equipment must be set considering the field conditions.

In the next section, the results of the application of the proposed method are presented. Initially, the results of different classifiers considering window sizes of 10, 50, and 100 records are presented. Then, the incorporation of Rocket, MiniRocket, and MultiRocket models with 10, 50, and 100 time steps are evaluated. Finally, the use of EMB methods to reduce noise that is not significant is evaluated.

## 5. Results

In the experiments presented in this section, a k-fold cross-validation methodology is used to evaluate the performance of the models, where k is equal to five. Cross-validation is a widely used technique to estimate the predictive performance of a model; in particular, 5-fold cross-validation involves splitting the dataset into five equal-sized partitions. Four partitions are used to train the model for each fold, and the remaining partition is utilized for testing. This procedure is repeated five times, such that each fold serves as the test set exactly once. The resulting accuracy scores from each fold are then averaged to estimate the model’s accuracy The default scikit-learn [116] parameters were employed in all the classification algorithms.

**Figure 5:**
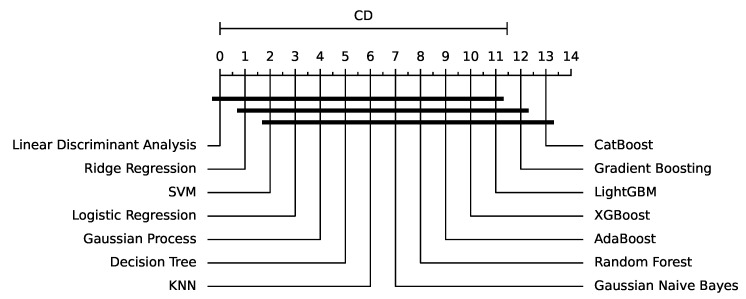
Critical Difference Diagram for the results of Table 1.

The performance of various algorithms for fault detection in insulators is evaluated using three different time window sizes, namely WS10, WS50, and WS100. Table 1 presents the accuracy results of 14 algorithms, including logistic regression, ridge regression, decision tree, k-NN, LDA, Gaussian Naive Bayes, SVM, random forest, gradient boosting, AdaBoost, Gaussian process, XGBoost, LightGBM, and CatBoost. The results indicate a clear trend regarding the time window size and the overall performance of the algorithms, with the critical difference diagram shown in Figure 5.

**Figure 6:**
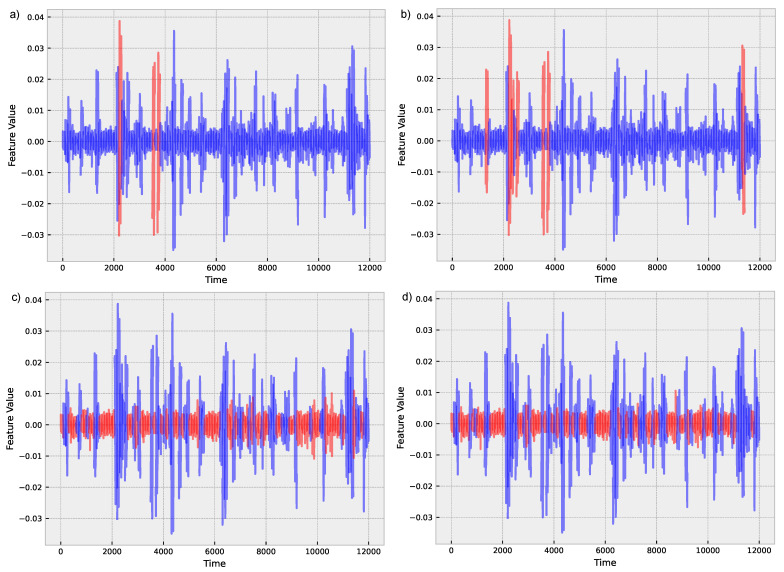
Classification for a time window of 100 for (**a**) Logistic Regression; (**b**) Ridge Regression; (**c**) Decision Tree; (**d**) XGBoost. Blue indicates normal operation conditions, while red indicates a fault.

From Table 1 it is evident that tree-based methods, such as decision tree, random forest, gradient boosting, AdaBoost, XGBoost, LightGBM, and CatBoost, exhibit superior performance compared to other algorithms, as can be further seen in Figure 6. CatBoost, LightGBM, and gradient boosting show the highest accuracies in WS50 and WS100 time windows, particularly strong results. Moreover, the table demonstrates that the accuracy of the algorithms generally improves as the time window size increases from WS10 to WS100. This observation suggests that longer time windows provide more information for the algorithms to identify the patterns and relationships between the features, resulting in improved performance. For instance, CatBoost’s accuracy increases from 0.8842 ± 0.0658 in WS10 to 0.95 ± 0.0459 in WS100, highlighting the significance of using longer time windows for fault detection in insulators.

Table 2, Table 3 and Table 4 present the results of different machine learning algorithms for insulator fault detection when using Rocket, MiniRocket, and MultiRocket data transforms for three different time windows: WS10, WS50, and WS100, respectively. These transforms were applied to enhance the time-series data and improve the performance of the algorithms. A notable outcome of these transformations is the improvement in accuracy across all algorithms, particularly in the case of linear algorithms.

Upon applying the Rocket, MiniRocket, and MultiRocket transforms, linear algorithms such as logistic regression, ridge regression, and LDA exhibit a substantial increase in their accuracy, as can be observed in the tables. These improvements can be attributed to the transforms’ ability to better capture the underlying patterns in the data, which allows linear algorithms to leverage this information and perform more effectively.

For instance, in Table 2, the accuracy of logistic regression increases from 0.7552 ± 0.0353 with Rocket to 0.8465 ± 0.06 with MultiRocket. In contrast, the accuracy of ridge regression increases from 0.6762 ± 0.0462 with Rocket to 0.8068 ± 0.0447 with MultiRocket. Similarly, in Table 3, logistic regression and ridge regression exhibit high accuracies of 0.955 ± 0.0395, and 0.9533 ± 0.036 with Rocket, respectively. These results suggest that the use of data transforms boosts the performance of linear algorithms, enabling them to compete with more complex models.

It is essential to note that the algorithm’s performance still improves as the time window size increases, consistent with the earlier observation in Table 1. This trend is evident across all three tables, reinforcing the importance of considering longer time windows when applying these algorithms to insulator fault detection.

### 5.1. Empirical Mode Decomposition

Table 5 presents the logistic regression results applied to fault detection in insulators with the MiniRocket transform for a window size of 100 (WS100), and further explores the impact of combining the MiniRocket transform with three different EMB methods: EWT, CEEMDAM, and VMD. The purpose of applying these EMB methods before the MiniRocket transform is to explore if the predictions can be further improved.

When these EMB methods are applied in conjunction with MiniRocket, the accuracy of logistic regression significantly improves compared to when using the MiniRocket transform alone. This improvement can be attributed to the ability of EMB methods to decompose the time series into different components, thereby highlighting the underlying patterns and structures in the data that may not be easily captured by the MiniRocket transform.

**Figure 7:**
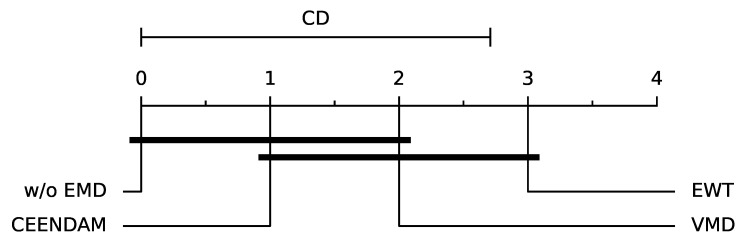
Diagram of Critical Difference for the results of Table 5.

For example, when using the EWT method, the accuracy of logistic regression increases from 0.9783 ± 0.0194 without EMB to 0.995 ± 0.0067 with EWT. Similarly, the accuracy of logistic regression improves to 0.9917 ± 0.0105 with CEENDAM and 0.98 ± 0.0187 with VMD. Figure 7 presents the critical difference diagram comparing the methods. These results indicate that applying EMB methods before the MiniRocket transform enhances the performance of logistic regression by providing a more refined representation of the data. In Table 6, the advantages and disadvantages of the classification methods are compared.

### 5.2. Discussion

The findings in the preceding subsections offer insightful information on how various machine learning techniques perform in defect detection. The study of these results can aid in the selection of acceptable methodologies and techniques for fault detection in insulators, notably the effects of time window size and data transform. The performance of the models was constantly enhanced by increasing the temporal window size. Longer time windows provide the algorithms with more information to find patterns and connections between features, improving insulator defect identification.

According to this study, larger time windows should be favored in practical applications to improve the precision of defect detection models. The advantages of extended periods must be weighed against the additional computational expenses. The amount of data being processed and the complexity of the models grow as the time window size grows. More extended training periods and increased computational demands may result from this. Therefore, when choosing the time window size for insulator fault detection, practitioners should carefully consider the trade-offs between the improvements in accuracy and the extra computational resources needed.

The results show that tree-based approaches, such as random forests, decision trees, gradient boosting, AdaBoost, XGBoost, and LightGBM, consistently outperform other algorithms in insulator fault detection. These techniques offer great accuracy when identifying insulator faults and are particularly good at managing non-linear connections between features. This shows that tree-based approaches should be the best options for insulator failure detection jobs. However, tree-based methods might be more prone to overfitting than other algorithms, particularly when working with small datasets. Pruning is one regularization approach that should be used to decrease overfitting risks and preserve model generalizability.

Logistic regression, ridge regression, and LDA perform much better when Rocket, MiniRocket, and MultiRocket data transforms are used. These modifications allow linear algorithms to take advantage of the information and perform better, even competing with more complicated models, by capturing more underlying patterns in the data. This result suggests that data transforms can be a useful preprocessing step in real-world applications, especially when using linear algorithms for insulator fault detection. Engineers may simplify their models by using these transforms while maintaining excellent fault detection accuracy.

Further, the results indicate that combining EMD methods and MiniRocket transform enhances the performance of logistic regression by providing a more refined representation of the data. This suggests that using EMD methods can improve fault detection capabilities when used in conjunction with rocket-like algorithms. The following guidelines can be offered for insulator failure detection using ultrasound signals in light of the study’s findings:

- Consider the trade-offs with computational resources and training timeframes carefully when using longer time windows to increase the fault detection models’ accuracy.
- Consider the use of tree-based algorithms for insulator failure detection, such as CatBoost, LightGBM, and gradient boosting, while being cautious of overfitting concerns and using regularization techniques as necessary. To improve the efficiency of linear algorithms and potentially reduce model complexity while retaining high accuracy, use data transforms like Rocket, MiniRocket, or MultiRocket.
- Employ EMD methods to enhance the performance of less complex regression methods by providing a more refined representation of the data and improving fault detection capabilities.

By using these suggestions, engineers can create more successful insulator failure detection models that improve the dependability and safety of electrical power systems. The next section presents the final remarks and suggestions for future work. The conclusions will be related to the applicability of ultrasound, the best structure to classify the time series, and what can be accomplished by the ultrasound equipment in future projects.

## 6. Conclusions and Future Research Directions

Using ultrasound as a decision-making support tool during inspections of the electrical power grid, combined with deep learning, proves to be promising since the proposed model achieves acceptable classification results. A significant advantage of using this equipment is that it is not necessary to measure contact with the electrical potential, reducing the risk to the operator and improving flexibility in inspecting the distribution power grid.

The findings indicate that tree-based methods, such as decision tree, random forest, gradient boosting, AdaBoost, XGBoost, and LightGBM, generally outperform other algorithms in terms of accuracy. Longer time windows (e.g., WS100) resulted in improved performance across all algorithms, suggesting that larger windows provide more information for pattern identification. Additionally, the application of Rocket, MiniRocket, and MultiRocket data transforms led to a significant increase in accuracy for linear algorithms such as logistic regression, ridge regression, and linear discriminant analysis. This improvement could be attributed to the transforms’ ability to capture the underlying patterns in the data better, enabling linear algorithms to perform more effectively.

In future work, it could be promising to evaluate the development of an embedded system to perform inspections based on the model presented in this paper. The evaluated model has a low computational effort in the test phase, making its application in an embedded system an interesting potential solution, in addition to the test performed with the aid of a personal computer. Considering the promising results achieved by the applied classifiers, in future work, more state-of-the-art models could be evaluated to obtain a broader analysis that could be applied to other components of the power grid.

## Figures and Tables

**Table 1:** Accuracy of different methods for windows size of 10, 50, and 100 (best results are in bold).

Model	WS10	WS50	WS100
Logistic Regression	0.5193 ± 0.0395	0.5167 ± 0.0325	0.5683 ± 0.0436
Ridge Regression	0.4923 ± 0.0134	0.5158 ± 0.0308	0.58 ± 0.041
Decision Tree	0.849 ± 0.0832	0.8658 ± 0.0789	0.8283 ± 0.0759
k-NN	0.8762 ± 0.0713	0.9025 ± 0.0748	0.85 ± 0.1182
LDA	0.4858 ± 0.0147	0.495 ± 0.0286	0.525 ± 0.0247
Gaussian Naive Bayes	0.8428 ± 0.0927	0.9133 ± 0.0746	0.9283 ± 0.0586
SVM	0.5343 ± 0.0379	0.5283 ± 0.0263	0.53 ± 0.0306
Random Forest	0.8672 ± 0.0815	0.9225 ± 0.0621	0.925 ± 0.0548
Gradient Boosting	**0.8792** ± 0.0694	**0.9433** ± 0.0439	0.9433 ± 0.0464
AdaBoost	0.8693 ± 0.07	0.9258 ± 0.0504	0.9317 ± 0.0593
Gaussian Process	0.6085 ± 0.0811	0.6342 ± 0.0564	0.615 ± 0.0883
XGBoost	0.8753 ± 0.0691	0.9417 ± 0.0484	0.935 ± 0.0539
LightGBM	0.8732 ± 0.0695	0.94 ± 0.0467	**0.95** ± 0.0431

**Table 2:** Accuracy of different methods with different rocket methods for a window size of 10 time steps (best results are in bold).

Model	Rocket	MiniRocket	MultiRocket
Logistic Regression	0.7552 ± 0.0353	0.8453 ± 0.068	0.8465 ± 0.06
Ridge Regression	0.6762 ± 0.0462	0.7943 ± 0.0518	0.8068 ± 0.0447
Decision Tree	0.7427 ± 0.0617	0.8635 ± 0.0687	0.8687 ± 0.064
k-NN	0.7375 ± 0.0387	0.8488 ± 0.0729	0.8623 ± 0.0676
LDA	0.6048 ± 0.0635	0.7832 ± 0.0421	D.N.C. *
Gaussian Naive Bayes	0.7615 ± 0.0515	0.8253 ± 0.0926	0.8342 ± 0.0894
SVM	0.6968 ± 0.0438	0.8257 ± 0.0647	0.8413 ± 0.0583
Random Forest	0.762 ± 0.0553	0.8788 ± 0.0659	0.882 ± 0.0676
Gradient Boosting	0.7735 ± 0.0543	**0.8837** ± 0.0655	**0.8873** ± 0.0632
AdaBoost	0.7452 ± 0.0544	0.8678 ± 0.0695	0.8715 ± 0.0639
XGBoost	**0.7623** ± 0.0472	0.8785 ± 0.0687	0.8823 ± 0.0638
LightGBM	0.7713 ± 0.0482	0.8832 ± 0.067	**0.8873** ± 0.0622

* Did not converge (D.N.C.).

**Table 3:** Accuracy of different rocket methods with a window size of 50 time steps (best results are in bold).

Model	Rocket	MiniRocket	MultiRocket
Logistic Regression	**0.955** ± 0.0395	**0.955** ± 0.0395	0.955 ± 0.0384
Ridge Regression	0.9533 ± 0.036	0.9533 ± 0.036	0.9508 ± 0.0389
Decision Tree	0.9258 ± 0.0551	0.9342 ± 0.0468	0.9367 ± 0.0511
k-NN	0.9483 ± 0.0427	0.9483 ± 0.0427	0.9433 ± 0.043
LDA	0.9533 ± 0.0361	0.9533 ± 0.0361	0.9492 ± 0.0418
Gaussian Naive Bayes	0.9308 ± 0.0491	0.9308 ± 0.0491	0.9283 ± 0.0502
SVM	0.9525 ± 0.0398	0.9525 ± 0.0398	0.9525 ± 0.0368
Random Forest	0.9483 ± 0.0459	0.9508 ± 0.0461	0.9483 ± 0.0402
Gradient Boosting	0.9517 ± 0.042	0.9483 ± 0.0452	0.9492 ± 0.0414
AdaBoost	0.9475 ± 0.0416	0.9475 ± 0.0416	0.955 ± 0.0349
Gaussian Process	0.9367 ± 0.0509	0.9367 ± 0.0509	D.N.C. *
XGBoost	0.9475 ± 0.044	0.9475 ± 0.044	0.9575 ± 0.0339
LightGBM	0.9542 ± 0.0365	0.9542 ± 0.0365	**0.9592** ± 0.0309

* Did not converge (D.N.C.).

**Table 4:** Accuracy of different methods with different rocket methods for a window size of 100 time steps (best results are in bold).

Model	Rocket	MiniRocket	MultiRocket
Logistic Regression	**0.9783** ± 0.0194	**0.9783** ± 0.0194	0.9733 ± 0.0249
Ridge Regression	0.9767 ± 0.0193	0.9767 ± 0.0193	0.9717 ± 0.034
Decision Tree	0.9633 ± 0.0323	0.9667 ± 0.0316	0.97 ± 0.0282
k-NN	0.9567 ± 0.037	0.9567 ± 0.037	0.9683 ± 0.0309
LDA	0.97 ± 0.0261	0.97 ± 0.0261	0.975 ± 0.0247
Gaussian Naive Bayes	0.945 ± 0.0515	0.945 ± 0.0515	0.9483 ± 0.0392
SVM	0.9783 ± 0.018	**0.9783** ± 0.018	0.9717 ± 0.0277
Random Forest	0.9717 ± 0.0314	0.9767 ± 0.0244	0.9733 ± 0.0309
Gradient Boosting	0.9683 ± 0.0271	0.97 ± 0.0251	0.9717 ± 0.0245
AdaBoost	**0.9783** ± 0.0201	0.9733 ± 0.0295	0.965 ± 0.0399
Gaussian Process	0.96 ± 0.0363	0.96 ± 0.0363	D.N.C. *
XGBoost	0.9767 ± 0.0249	0.9767 ± 0.0249	**0.975** ± 0.0228
LightGBM	0.9767 ± 0.022	0.9767 ± 0.022	0.965 ± 0.0429

* Did not converge (D.N.C.).

**Table 5:** Results for logistic regression considering different window sizes and EMB.

	Accuracy			
**Window Size**	**W/o EMB**	**EWT**	**CEENDAM**	**VMD**
10	0.8487±0.0711	0.9757±0.0177	0.9192±0.129	0.9677±0.0267
50	0.9583±0.0333	0.9883±0.0116	0.98±0.0278	0.9767±0.0187
100	0.9783±0.0194	0.995±0.0067	0.9917±0.0105	0.98±0.0187

**Table 6:** Comparison with other approaches.

Author	Approach	Advantages	Disadvantages
[6]	EN-ELM	Computation is fast.	It might obtain the wrong measurements because of interference.
[39]	Pseudo-prototypical part network.	It has interpretable results.	If the data are not correctly selected in the first step, the model will not work.
[41]	Hybrid-YOLO	It obtains a better performance than standard approaches.	If the data are not correctly selected in the first step, the model will not work.
[63]	ESN	It excels for drilling classification.	It has lower accuracy for multiclassification.
Our Method	Rocket with EMD	It is adaptable.	Needs an operator to set the ultrasound equipment.

## Data Availability

Data can be provided upon request.
